# The silence of MUC2 mRNA induced by promoter hypermethylation associated with HBV in Hepatocellular Carcinoma

**DOI:** 10.1186/1471-2350-14-14

**Published:** 2013-01-25

**Authors:** Yang Ling, Jing Zhu, Lu Gao, Yongping Liu, Changtai Zhu, Rong Li, Lixin Wei, Changsong Zhang

**Affiliations:** 1 Clinical Oncology Laboratory, Changzhou Tumor Hospital, Soochow University, Changzhou, No.1 North Huaide Road, Changzhou, 213001, China; 2 Tumor Immunology and Gene Therapy Center, Eastern Hepatobiliary Surgery Hospital, the Second Military Medical University, Shanghai, China

**Keywords:** MUC2, Hepatocellular carcinoma, Methylation

## Abstract

**Background:**

To evaluate the promoter methylation status of MUC2 gene and mRNA expression in patients with hepatocellular carcinoma.

**Methods:**

We analyzed MUC2 methylation by MSP, and MUC2 mRNA by real-time PCR in 74 HCC.

**Results:**

MUC2 mRNA were lower in HCC tissues (Mean _-ΔCt_ = −4.70) than that in Non-HCC tissues (Mean _-ΔCt_ = −2.98). Expression of MUC2 was elevated in only 23 (31.08%) of the 74 HCC patients. MUC2 promoter was hypermethylated in 62.2% (46/74) of HCCs, and in only 18.9% (14/74) of non-tumor samples. MUC2 mRNA were lower in HCC patients with hypermethylation (Mean _-ΔΔCt_ = −2.25) than those with demethylation (Mean _-ΔΔCt_ = −0.22), and there is a decreased tendency for MUC2 mRNA in HCC patients with promoter hypermethylation (*p* = 0.011). There was a significantly correlation found between MUC2 mRNA and HBV and AFP in HCC. The loss of MUC2 mRNA and hypermethylation could be poor prognostic factors. After treated by 5-Aza-CdR and TSA, we found that MUC2 mRNA induced significantly in 7721, Huh7 and HepG2 cells.

**Conclusion:**

The results suggested that MUC2 mRNA silenced by promoter hypermethylation is associated with high levels HBV in HCC.

## Background

Mucins are high molecular weight glycoprotein components of mucus, which protect and lubricate the epithelial surfaces of the respiratory, gastrointestinal and reproductive tracts in the body. In humans, to date, about six secreted and 14 membrane-tethered mucins have been reported based on cloned complementary DNA (cDNA) sequences [1,2].

MUC2 is the major secreted mucin in the large and small intestine with an O-linked carbohydrate. MUC2 presents in normal gastrointestinal secretion products and epithelia, and in some tumors [3]. Alteration of MUC2 expression may contribute to change in growth regulation, immune recognition, cellular adhesion, carcinoma-host and other cellular interactions, which may influence the invasive and metastatic capabilities of the cancer [4,5]. The aberrant expression of MUC2 is together with altered expression of MUC5AC and MUC6 in intestinal metaplasia during the process of gastric carcinogenesis. And the MUC2 expression pattern is a reliable marker of intestinal metaplasia associated *H. pylori* infected individuals [6]. The increased MUC2 expression in intestinal metaplasia in the neighborhood of the carcinomas may play an important role in gastric carcinomas or IPMN [7,8]. It has been recently suggested that mucin genes have a regulatory role for their products during cell proliferation and differentiation, and this leads to carcinogenesis when these gene products are expressed inappropriately in the pathogenesis of breast cancer, gastric carcinomas, etc. [1,9].

Human normal bile ducts do not show MUC2, and MUC2 mRNA was detectable in the normal cholangiocytes. But the presence of MUC2 protein was not demonstrable by immunohistochemical staining cholangiocarcinoma [10,11]. MUC2 expression were observed in 42.0% of 193 extrahepatic bile duct carcinomas [12]. The conventional intrahepatic cholangiocarcinoma (ICC) frequently expressed MUC5AC, but no MUC2, in carcinoma cells. Therefore, the expression of MUC2 seems to be a specific feature of mucinous ICC and Intraductal papillary neoplasia of the liver [13]. Colonic epithelial metaplasia resembling regenerating colonic epithelia and/or tubular adenoma of the colon also occur in hyperplastic and dysplastic biliary lining epithelia in chronic biliary disease and is positive for MUC2 and CK20 [14,15]. However, the results regarding correlations of MUC2 expression in cancer are contradictory [16].

Given that the aberrant expression of MUC2, it is conceivable that MUC2 may be also involved in the development of cellular differentiation in Hepatocellular Carcinoma [17]. Relatively little is known, however, about the mechanisms responsible for regulation of MUC2 expression in HCC. MUC2 gene regulation mechanism disclosed that DNA methylation and histone modification in the 5’ flanking region of the *MUC2* promoter may play an important role [4]. MUC2 are highly submitted to DNA methylation and histone modifications, and MUC2 repression by cell-specified methylation is controlled by DNA methyltransferase 1 (DNMT1) and dramatically impairs their activation by the transcription factor Sp1 in epithelial cancer cells [18,19]. MUC2 expression in gastric cells is regulated by promoter methylation with two specific CpG sites [20]. And the low methylation status of MUC2 gene plays a predominant role in high level MUC2 expression in mucinous colorectal cancer [21]. The histone H3 modification could play an important role in MUC2 gene expression, possibly affecting DNA methylation in pancreatic neoplasm [22]. It implied that the promoter methylation of MUC2 could play a particularly important regulatory role for MUC2 expression in carcinogenesis.

So far the few studies conducted focused on MUC2 methylation and no data are available regarding MUC2 in HCC. In this study, we examined the expression of MUC2 with respect to the promoter methylation in HCC.

## Materials and methods

### Patients and tissue samples

All of these cases were surgically resected from 74 patients with HCC, and were obtained from our departments and affiliated hospitals. The tissues samples were flash frozen in liquid nitrogen immediately after surgical resection. The matched non-HCC tissues were obtained from the liver >3 cm away from tumors and were confirmed to be tumor-free by microscopic examination. The patients consisted of 65 men and 9 women, ranging in age from 27 to 70 years (mean ± SD, 49.51 ± 11.12 years). All tumors were histologically diagnosed as HCC according to the Edmondson-Steiner classification system [23]. Written informed consent was obtained from each patient, and the protocol of the study was approved by the local ethics committee of Soochow University.

### Cell culture and treatment

The HCC cancer cell lines (7721, Huh7 and Hep-G2) were kept in our laboratory. The cells were cultured in RPMI medium plus 10% fetal bovine serum in a humidified 37°C incubator containing 5% CO2. They were plated and treated with final concentration of 10 μM 5-Aza-2’-deoxycytidine (5-Aza-CdR, Sigma, St. Louis, MO, USA) and 400 ng/ml Trichostatin A (TSA, Sigma, St. Louis, MO, USA). The fresh medium was changed every 24 hours to maintain the 5-Aza-CdR and TSA concentration. RNA was isolated 3 days after treatment. DMSO was being a blank control.

### Methylation analysis of MUC2

The bisulfite modification of DNA was done according to described previously [24]. MUC2 methylation was measured using a methylation-specific PCR assay as previously described [22]. Primers used were: unmethylated MUC2 forward primer, 5’-TTATATAAGTTAGTGGTTTTTTTGG-3’, reverse primer, 5’-AATCTAATCAAACTCCTTAACCCAC-3’ (217 bp); methylated MUC2 forward primer, 5’-TTTATATAAGTTAGTGGTTTTTTCGG-3’, reverse primer, 5’-AATCTAATCAAACTCCTTAACCCG-3’ (216 bp). The PCR products were separated on 2% agarose gels and visualized using ethidium bromide staining. The methylation index (MI) of MUC2 was calculated by the following formula: 100 × methylated reaction/(unmethylated reaction + methylated reaction). ΔMI defined as MI_HCC_ - MI_Non-HCC_. Distilled water was used as negative control, DNA methylated by SssI methylase (Sss DNA) was used as positive control.

### Quantitative real-time PCR analysis for MUC2

Total RNA was isolated from 74 HCC, adjacent normal tissues, and cultured cells. The first-strand cDNA was synthesized from 2 μg of total RNA. Primer sequences of MUC2 for reverse transcription-PCR (RT-PCR) reaction were forward (5’- CTTCGACGGACTCTACTACAGC-3’) and reverse (5’- CTTTGGTGTTGTTGCCAAAC-3’) [25]. Quantitative real-time PCR (qPCR) were carried out by using the M×3000P QPCR System (Stratagene, California, USA). The cDNA was then used for qPCR in a 20 μl SYBR Premix Ex Taq. qPCR for MUC2 mRNA expression was performed under the following conditions: 5 min at 95°C, 40 cycles of 30 seconds at 95°C, 30 seconds at 60°C, and 1 min at 72°C. As an internal control for qPCR, β-actin mRNA expression was amplified from the same cDNA samples. All results were normalized to β-actin amplification. *C*_T_ values for triplicate reactions were averaged and relative MUC2 expression was determined with the comparative *C*_T_ method, using average *C*_T_ values for MUC2 and β-actin.

### Statistical analysis

All data were generated without knowledge of the clinical status of the samples analyzed by SPSS 17.0 software (SPSS, Inc., Chicago, USA). Associations between categorical variables were examined by using the Pearson χ^2^ and Fisher exact tests. Kaplan–Meier analysis and the log rank test were performed to identify survival differences in HCC. A *P* value of less than 0.05 was considered statistically significant.

## Results

### The levels of MUC2 mRNA in HCC and corresponding non-tumor tissues

**Figure 1:**
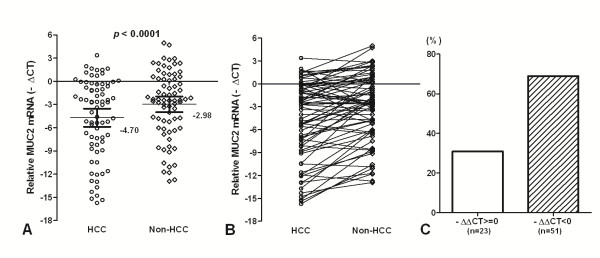
**MUC2 mRNA in HCC and the matched non-tumor liver tissues.** (**A**) The MUC2 mRNA was indicated in HCC and Non-HCC samples. Data are shown by the Mean _-ΔCT_ and 95%CI. The MUC2 mRNA in HCC was lower than that in the matched non-tumor liver tissues (*p* < 0.0001). (**B**) There was a decreased tendency for MUC2 expression from Non-HCC tissues to HCC. (**C**) Expression of MUC2 was elevated (−ΔΔCt > =0) in only 23 of the 74 HCC patients but decreased (−ΔΔCt < 0) in 51 of the patients. Statistical analyses were done using the paired *t* test.

To accurately quantify relatively MUC2 mRNA levels, we used a real-time PCR assay in 74 HCC and matched non-tumor tissues. Overall results of MUC2 mRNA are summarized in Figure 1. We found that MUC2 mRNA expression lower in HCC tissues (Mean _-ΔCt_ = −4.70; 95% CI, -5.88 – -3.53) than that in Non-HCC tissues (Mean _-ΔCt_ = −2.98; 95% CI, -3.99 – -1.97). MUC2 expression was significantly difference between HCC tissues and matching non-tumor tissues (*p* < 0.0001; Figure 1A). There was a decreased tendency for MUC2 expression from Non-HCC tissues to HCCs, and more HCC samples showed lower MUC2 expression (Figure 1B). Expression of MUC2 was elevated (−ΔΔCt > =0) in only 23 (31.08%) of the 74 HCC patients but decreased (−ΔΔCt < 0) in 51 (68.92%) of the patients (Figure 1C). This would suggest that the loss of MUC2 gene expression is a critical requirement for the development of HCC.

### Association of MUC2 mRNA with clinicopathologic features

The relationship between MUC2 mRNA status and known clinicopathologic factors in 74 tumor tissues were examined. Initially analyzed were the associations between mRNA status and available clinical information including age, gender, differentiation of the tumor, presence of hepatitis, presence of cirrhosis, tobacco, alcohol, AFP. These analyses were summarized in Table 1. Significantly, the lower MUC2 mRNA was found in HCC patients with HBV > = 10^5^ (copy/ml) than those with HBV < 10^5^ (copy/ml) (Mean _-ΔCt_ ± SE, -6.69 ±1.08 and −3.69 ± 0.67, respectively) (p = 0.015). Meanwhile, the MUC2 mRNA was decreased in tumor tissues with age > = 40 years than those with age < 40 years in HCC patients (Mean _-ΔCt_ ± SE, -5.57 ± 1.89 and −1.29 ± 0.80, respectively) (p < 0.001). But the MUC2 mRNA was elevated in tumor tissues with AFP > = 30 (μg/l) than those with AFP < 30 (μg/l) in HCC patients (Mean _-ΔCt_ ± SE, -3.73 ± 0.75 and −6.25 ± 0.94, respectively) (p = 0.040). There was no other significant correlation found between other clinicopathological factors and MUC2 mRNA in Chinese HCC. These results implicated that HBV and age could play an important role for the loss of MUC2 gene expression in HCC.

**Table 1:** Correlation of clinicophthologic variables with MUC2 mRNA in HCC

**Variable**		**No.(74)**	**Mean**_**-Δct**_**(± SE)**	*P *value*
Gender	Male	65	−4.63 (± 0.62)	0.722
	Female	9	−5.27 (± 1.89)	
Age(y)	> = 40	59	−5.57 (± 0.67)	**< 0.001**
	< 40	15	−1.29 (± 0.80)	
HBV history	Yes	43	1.07 (± 0.65)	0.393
	No	31	1.89 (± 0.68)	
Differentiation	G_1_	62	−4.61 (± 0.65)	0.733
	G_2-3_	12	−5.17 (± 1.44)	
Cirrhosis	Yes	39	−4.22 (± 0.82)	0.391
	No	35	−5.24 (± 0.86)	
Tobacco	Yes	33	−5.04 (± 0.88)	0.609
	No	41	−4.43 (± 0.80)	
Alcohol	Yes	14	−4.01 (± 1.32)	0.572
	No	60	−4.87 (± 0.66)	
HBV (copy/ml)	> = 10^5^	25	−6.69 (± 1.08)	**0.015**
	< 10^5^	49	−3.69 (± 0.67)	
AFP (μg/l)	> = 30	45	−3.73 (± 0.75)	**0.040**
	< 30	29	−6.25 (± 0.94)	

* Statistical significance determined using the independent samples test.

### Methylation status of MUC2 promoter in HCC and its adjacent tissue

**Figure 2:**
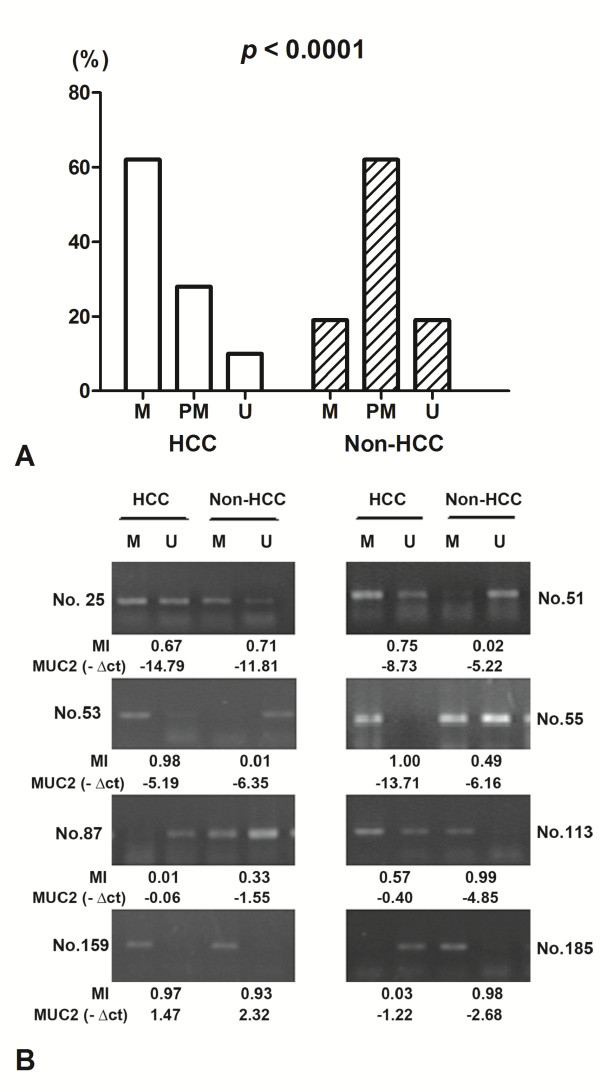
**The promoter methylation of MUC2 in HCC tissues and non-tumor tissues by MSP.** (**A**) Methylation of MUC2 promoter in HCC and corresponding non-carcinoma tissues. M, hypermethylation; PM, partial methylation; U, unmethylation; (**B**) Representative patterns of MUC2 promoter methylation. U, reaction specific for unmethylated DNA; M, reaction specific for methylated DNA. MI, The methylation index. -ΔCt, - (CT_MUC2_ - CT_β-actin_).

The methylation status of MUC2 promoter region was analyzed as one of the putative regulatory mechanisms of MUC2 mRNA expression in HCCs and their adjacent normal tissues. The hypermethylation contains only methylated PCR product, the partial methylation contains both methylated and unmethylated PCR products, and the unmethylation contains only unmethylated product. MUC2 promoter was hypermethylated in 62.2% (46/74) of HCCs, and in 18.9% (14/74) of non-tumor samples; partial methylated in 28.4% (21/74) vs. 62.2% (46/74); unmethylated in 9.4% (7/74) vs. 18.9% (14/74). The difference of MUC2 methylation between the tumor and non-tumor groups was statistically significant (p < 0.0001) (Figure 2A).

### Association of MUC2 methylation with MUC2 mRNA expression in HCC and corresponding normal tissues

**Figure 3:**
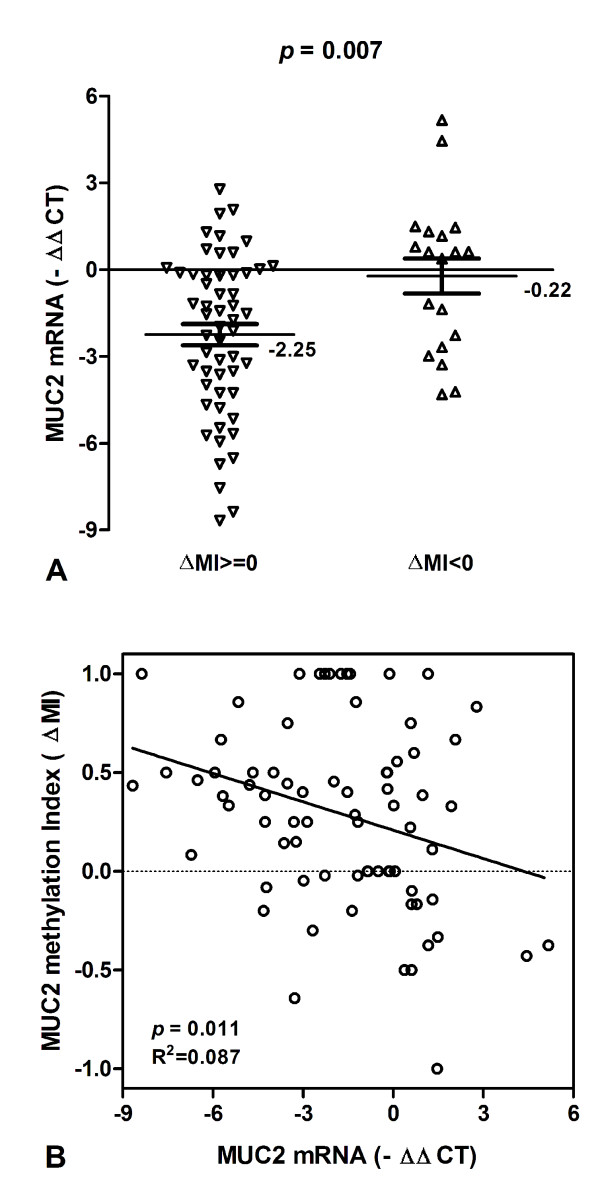
**MUC2 mRNA and promoter methylation in HCC patients.** (**A**) MUC2 mRNA expression were lower in HCC patients with ΔMI > = 0 than those with ΔMI < 0. Statistical analyses were done using the unpaired *t* test. (**B**) The scatter plots summarized relative expression levels of MUC2 mRNA (−ΔΔCT) associated with relative methylation index (ΔMI) in HCC patients. Statistical analyses were done using Pearson correlation test. -ΔΔCT, - (ΔCT_HCC_-ΔCT_Non-HCC_); ΔMI, MI_HCC_-MI_Non-HCC_.

To test whether MUC2 promoter methylation in HCC might be correlated with repression of MUC2 mRNA transcription, qPCR was used for the expression of MUC2 transcripts in all tissue samples (Figure 2B). The levels of MUC2 mRNA expression were significantly decreased in HCC samples with methylation (ΔMI > =0) than in those with hypomethylation (ΔMI < 0) (Mean _-ΔCt_ ± SE, -2.25 ± 0.37 and −0.22 ± 0.61, respectively; p = 0.007; Figure 3A). We found that MUC2 methylation is correlated significantly with MUC2 mRNA expression, and there is a decreased tendency for MUC2 mRNA in HCC patients with promoter hypermethylation (R^2^ Linear = 0.087, P = 0.011; Figure 3B). The results suggested that HCC showing hypermethylation of MUC2 promoter is considered to be silencing MUC2 mRNA expression.

### The survival analysis associated with MUC2 mRNA and methylation in HCC

**Figure 4:**
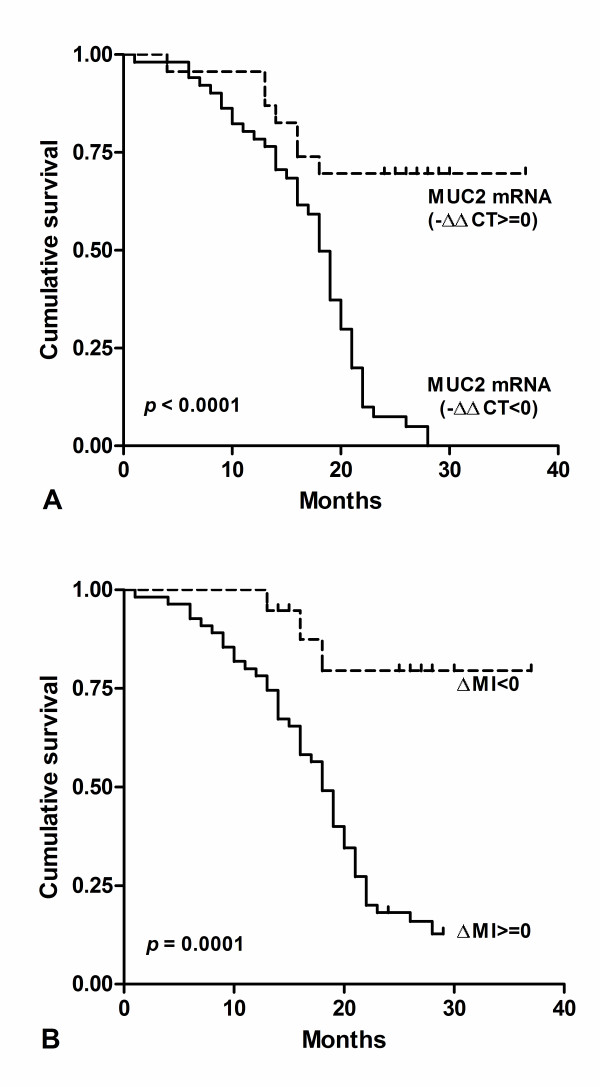
**MUC2 mRNA and hypermethylation confers poor prognosis in HCC.** (**A**) Kaplan–Meier analysis of survival months after surgery according to MUC2 mRNA level. (**B**) Kaplan–Meier analysis of survival months after surgery according to MUC2 methylation index.

The survival of these patients was compared by the Kaplan–Meier method and the log rank test (Figure 4). The MUC2 mRNA and promoter methylation was significantly correlated with overall survival after surgery. We found the decreased Expression of MUC2 (−ΔΔCt < 0) were significantly correlated with poor overall survival (*p* < 0.0001; HR = 0.238, 95% CI: 0.13-0.43; Figure 4A). Results showed the cumulative survival after surgery in HCC with ΔMI > =0 was significantly shorter than those with ΔMI < 0 (*p* = 0.0001; HR = 3.404, 95% CI: 1.81-6.39; Figure 4B). These results suggested that MUC2 mRNA and methylation level could be prognostic factors in HCC.

### MUC2 mRNA by 5-Aza-CdR and TSA

**Figure 5:**
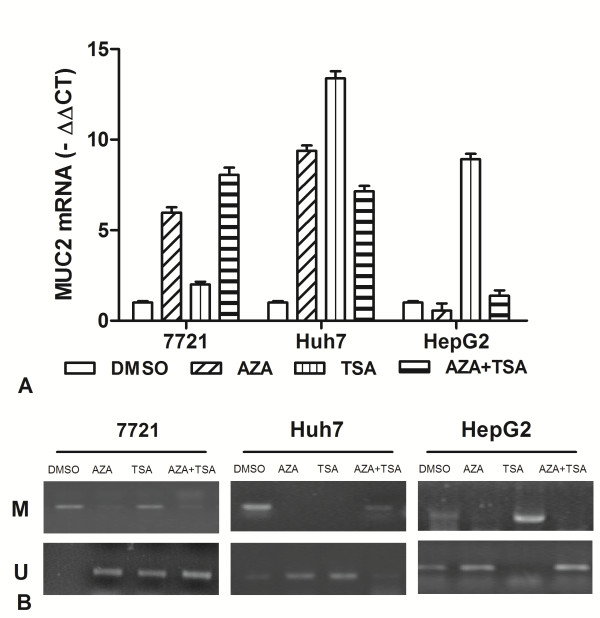
**Effect of 5-aza-CdR and TSA on expression of MUC2 transcription in HCC cell lines.** (**A**) The MUC2 mRNA was detected by real-time PCR for 7721, Huh7, and HepG2 treated with 5-aza-CdR, TSA, alone or combination. (**B**) It indicated that MUC2 demethylation induced significantly by TSA or TSA in 7721, Huh7 and HepG2 cells.

To analyze the effects of epigenetic inhibitor on MUC2 gene expression, Real-time PCR analyses were performed using HCC cancer lines (7721, Huh7 and Hep-G2) treated with final concentration of 10 μM 5-Aza-CdR and 400 ng/ml TSA. After normalizing mRNA levels to β-actin, a 5.9-9.4 ΔCt induction of MUC2 mRNA was detected after 5-Aza-CdR treatment in 7721 and Huh7 cells, but no change for Hep-G2 cells (Figure 5A). Additionally, qRT-PCR assays found that the expression of MUC2 gene was induced 2–13.4 ΔCt after TSA treatment in three cells. For the 5-Aza-CdR + TSA treatment, we found that a 7–8 ΔCt induction of MUC2 mRNA was detected in 7721 and Huh7 cells. Taken together, the above results suggested that the expression of MUC2 can be activated by 5-Aza-CdR or TSA, and the effect on MUC2 expression is very various for different cells.

Meanwhile, we observed the effects of 5-aza-CdR and TSA on promoter methylation of MUC2 gene by MSP. According to MSP analysis, the MUC2 promoter was found to be hypermethylated in 7721 and Huh7, but partial methylation in HepG2 cells (Figure 5B). The demethylation of MUC2 was found by 5-aza-CdR or TSA treatment in three cells. However, it showed different effects on MUC2 methylation. These data suggested the demethylation of MUC2 promoter by epigenetic inhibitor could play an important role for reactivating gene expression.

## Discussion

MUC2, as a secretory mucin with a central region composed of a tandem repeat sequences of 23 amino acids each, plays an important role for a physiological barrier against various aggressions of the underlying epithelia [26]. Given the putative role of MUC2 in tumourigenesis, understanding the mechanisms that regulate its activity is critical for a complete understanding of MUC2 function in HCC.

The results confirmed that MUC2 was lower expression in HCC tissues than corresponding normal tissues by Real-time PCR. Meanwhile, 23 of HCC patients were elevated for MUC2 mRNA, and 51 cases were decreased for MUC2 mRNA. MUC2 mRNA was a statistically significant difference between HCC and non-HCC tissues. However, the loss of MUC2 mRNA could play more complex role in the pathogenesis of HCC. The regulatory mechanism of the MUC2 gene is unclear. We found that the frequency of MUC2 hypermethylation was 46 cases in HCC samples and 14 cases in non-tumor tissues. The MUC2 mRNA expression were significantly decreased in HCC samples with hypermethylation (ΔMI > =0) than in those with hypomethylation (ΔMI > =0). It was a decreased tendency for MUC2 mRNA in HCC patients with promoter hypermethylation. The results suggested that HCC showing hypermethylation of MUC2 promoter is considered to be silencing MUC2 mRNA expression. This study showed that MUC2 expression could be regulated by DNA methylation in the promoter region in HCC.

We found that there is a significantly correlation found between MUC2 mRNA and HBV and AFP in HCC. In particular, the decreased expression of MUC2 and hypermethylation clearly identified patients with a poorer prognosis. One possible explanation could be that high level of HBV virus is an important factor to regulate methylation of MUC2 promoter in hepatocarcinogenesis. HBx was positively correlated with the DNMT1 and DNMT3A at both the mRNA and protein level [27]. And HBx expression could up-regulate DNMT1, DNMT3A1, and DNMT3A2 activities and selectively promoted hypermethylation of specific tumor suppressor genes [28]. But the reason in term of MUC2 hypermethylation is not yet well understood.

Epigenetic is essential for not only the maintenance but also the initiation of many tumor types. The epigenetic inhibitors 5-Aza-CdR or TSA play an important role for regulating transcriptional activity of related gene [29]. Quantitative RT–PCR analysis of HCC cells showed that treatment with 5-Aza-CdR or TSA gave a different change in MUC2 mRNA. The 5-Aza-CdR alone treatment was more effective in 7721 and Huh7 than Hep-G2. The TSA alone treatment was more effective in Huh7 and Hep-G2 than 7721. And the combination treatment was more effective for 7721 and Huh7 than Hep-G2 in increasing MUC2 mRNA. Meanwhile, we observed the different effects of epigenetic inhibitors on promoter demethylation of MUC2 gene in three cells. The combination treatment in Huh7 showed a little demethylation, which could be due to individual differences of cancer cells by incubated with 5-Aza-CdR and TSA together. The inhibitors of histone deacetylation and DNA methylation could have a different synergistic effect of MUC2 mRNA on cancer cells. These results suggested that DNA epigenetic modification influence MUC2 gene expression.

## Conclusions

MUC2 promoter hypermethylation is frequently observed in HCC and is associated with loss of mRNA expression and loss of MUC2 mRNA and promoter hypermethylation is significantly correlated with worse survival in HCC. There was a significantly correlation found between MUC2 mRNA and HBV and AFP in HCC. An understanding of these intimately correlated epigenetic changes may be of importance for predicting the outcome of patients with MUC2. Further investigations regarding the role of MUC2 expression in HCC are necessary.

## Competing interests

The authors declare that they have no competing interests.

## Authors' contributions

LR and GL carried out the majority of the cellular and molecular studies, participated in drafted the manuscript. ZJ, ZCT, and LYP participated in qRT-PCR assay. LY and WLX participated in result analysis and helped to draft the manuscript. ZCS conceived of the study, and participated in its design and coordination and helped to draft the manuscript. All authors read and approved the final manuscript.

## Pre-publication history

The pre-publication history for this paper can be accessed here:

http://www.biomedcentral.com/1471-2350/14/14/prepub
